# MixRformer: Dual-Branch Network for Underwater Image Enhancement in Wavelet Domain

**DOI:** 10.3390/s25113302

**Published:** 2025-05-24

**Authors:** Jie Li, Lei Zhao, Heng Li, Xiaojun Xue, Hui Liu

**Affiliations:** Faculty of Information Engineering and Automation, Kunming University of Science and Technology, Kunming 650504, China or 20222204196@stu.kust.edu.cn (J.L.); liheng@kust.edu.cn (H.L.); xiaojun35678@kust.edu.cn (X.X.); liuhui621@126.com (H.L.)

**Keywords:** underwater image enhancement, wavelet transform, convolutional neural network, Transformer, multi-branch structure

## Abstract

This paper proposes an underwater image enhancement model MixRformer that combines the wavelet transform and a hybrid architecture. To address the problems of insufficient global modeling in existing CNN models, weak local feature extraction of Transformer and high computational complexity, multi-resolution feature decomposition is performed through a discrete wavelet transform (IWT/DWT) in which low-frequency components retain structure and texture, and high-frequency components capture detail features. An innovative dual-branch feature capture module (DFCB) is designed as follows: (1) the surface information extraction block combines convolution and position encoding to enhance local modeling; (2) the rectangular window gated Transformer expands the receptive field through the convolution gating mechanism to achieve efficient global relationship modeling. Experiments show that the model outperforms mainstream methods in color restoration and detail enhancement, while optimizing computational efficiency.

## 1. Introduction

As an information carrier for understanding and perceiving the underwater environment, underwater images play an important role in marine resource exploration, marine biodiversity research, underwater rescue, target identification, real-time tracking, etc. However, underwater images are often affected by light absorption and scattering caused by particles in the water, which can significantly reduce the color and contrast of underwater images. In addition, light of different wavelengths can also affect the quality of imaging. Due to the poor underwater imaging environment, color casts, color artifacts, and blurred details are common, even in high-end cameras. Underwater image enhancement technology was invented to improve the quality of underwater images. Therefore, the use of underwater image enhancement technology to obtain clear underwater images plays a vital role in various fields such as scientific research and resource development. Existing underwater image enhancement methods are generally divided into three categories: methods based on traditional physical models, methods based on visual priors, and methods based on deep learning.

Traditional physical models can enhance underwater images of specific scenes. He, K., Sun, J., & Tang, X et al. [1] proposed a single-image dehazing method based on dark channel prior, which effectively removed the haze of underwater images by simulating the atmospheric scattering model. Drews, P., Nascimento, et al. [2] proposed an algorithm for estimating underwater image transmission using the atmospheric scattering model, which effectively improved the clarity and contrast of the image. Although the physical model-based method can restore the appearance of underwater images, it relies on specific parameter estimation and cannot distinguish between texture detail information and noise in the image. While improving image details, it also inevitably increases noise. For images with uneven lighting, fog, high noise, etc., local areas are prone to over-enhancement, while background areas are prone to under-enhancement. The visual prior-based method ignores the physical model and improves the image visual quality by adjusting the image pixel values of contrast, brightness and saturation to comprehensively evaluate the image quality. Zhang et al. [3] proposed an enhancement method based on Retinex theory. By applying filters to each component in the Lab color space, they successfully eliminated noise and halo artifacts in the image, significantly improving the image quality. Drews, P., Nascimento, E., et al. [4] proposed an image restoration method based on depth estimation, which removes turbidity and improves image quality by estimating the depth of underwater scenes. Although it has strong adaptability and robustness and can provide consistent results in complex environments, its effect may be limited by specific prior knowledge or statistical characteristics, and may not perform as expected in low-contrast or high-noise images, resulting in artifacts, color deviation, oversaturation, and other problems in the generated images.

In recent years, more and more researchers have begun to build different deep learning model structures and have achieved good results and high efficiency. Most of the underwater image enhancement methods based on deep learning [5] are centered on end-to-end models and use pairs of degraded underwater images and high-quality corresponding images to train the model to effectively enhance the image contrast and correct the image color. However, after a large number of experiments on challenging datasets, we found that some methods often lead to blurred images or introduce unrealistic color changes. This is because the model ignores certain information under its powerful correction ability. Convolutional neural network [6] is the most classic network in computer vision. Underwater images are enhanced using a convolutional neural network. Although convolutional networks can effectively achieve high-precision local enhancement, it is difficult for convolution to model global long-range relationships, which seriously limits the development of underwater image enhancement tasks. To address the above problems, some articles use the self-attention module of Transformer [7] to expand the model’s ability to capture global long-range relationships. However, although Transformer has shown excellent performance in visual tasks, it lacks effective inductive bias and cannot encode the two-dimensional position information of the image. It needs to embed relative or absolute position encoding, which may lead to its encoding and generalization capabilities being inferior to convolutional networks. We believe that there are four reasons for this weakening. First, Transformer can effectively handle the dependencies between tokens, but its complexity is quadratic with the resolution of the image, that is, ${O((HW))}^{2}$, which can easily lead to insufficient GPU memory, low computational efficiency, and long training time. Secondly, due to the lack of interaction between windows in the square window of Transformer, the receptive field increases slowly, which affects the capture and processing of global information. In addition, the traditional feedforward layer MLP in Transformer is mainly used to process the information interaction of feature dimensions. Although the structure is simple and universal, the existence of GELU will truncate some data, resulting in the loss of key information, and more importantly, MLP cannot actively select which information needs to be retained or suppressed. Finally, Transformer mainly relies on global attention, cannot encode the two-dimensional position information of the image, and is difficult to model local information, resulting in a lack of local receptive fields and insufficient attention to details.

In order to solve the above problems, this paper proposes a dual-branch underwater image enhancement algorithm, MixRformer, that integrates CNN and Transformer in the wavelet domain. It is based on the U-Net structure. The convolutional layer of the original U-Net is replaced by a dual-branch feature extraction module (DFCB) for local and global interaction of multi-scale features and combined with jump connections to improve feature fusion and spatial information recovery.

In order to reduce the computational complexity of the Transformer and improve the inference speed, we introduce a wavelet transform (DWT-IWT) for up-sampling and down-sampling [8], which reduces the resolution while retaining key features. In addition, inspired by CAT [9], we propose the Rectangle GLU-Window Transformer module, which combines horizontal and vertical rectangular window attention mechanisms to expand the receptive field without increasing the computational complexity and enhances the information interaction between windows through Axial-Shift. The feedforward network is also crucial for the Transformer. We use ConvGLU instead of MLP, introduce a gating mechanism to dynamically adjust channel activation, selectively retain or suppress information, and use convolution instead of SE modules to enhance local feature modeling ability. Given that images are different from sequence data and have a two-dimensional spatial structure, the Transformer is less efficient in extracting local features (such as edges, textures) and encoding two-dimensional information. Compared with the recent CNN-Transformer model, we first introduced the wavelet transform into a dual-branch structure for underwater enhancement tasks. At the same time, on the basis of reducing the computational complexity and parameter quantity, we combined the frequency domain information to synergistically improve various indicators of underwater images. Therefore, combining the advantages of CNN and Transformer is an effective way to improve the image enhancement effect.

In general, the unique contributions of this paper are summarized as follows:

1. This work proposes, for the first time, an underwater image enhancement algorithm structure, MixRformer, that effectively combines the wavelet transform with CNN-Transformer. The introduction of the wavelet transform is not only conducive to restoring the detailed features of the image, but also can reduce the image resolution, thereby reducing GPU memory consumption and reducing the amount of calculation of Transformer, so that MixRformer can better restore the color and texture of the image.
2. We propose a dual-branch feature capture block (DFCB), which consists of a simple surface information extraction block, ConvBlock, and an innovative Rectangle GLU-Window Transformer block, which are used to extract surface details and capture global features, respectively.
3. We construct a multi-loss function and introduce MSE Loss and VGG Perceptual Loss to train the model for restoring underwater distorted images. Compared with several state-of-the-art underwater image enhancement (UIE) methods, our proposed method shows extremely excellent performance in both objective indicators and visual quality. In particular, it has significant advantages in eliminating color casts and improving image clarity.

## 2. Related Works

### 2.1. Underwater Image Restoration Task

Underwater image restoration is a long-standing low-level vision problem that aims to restore high-quality images from low-quality images. In recent decades, many researchers have proposed various theories and methods to improve the quality of image restoration in order to solve these challenging tasks. For example, 1. Hou et al. proposed a variational model based on non-local micromolecules, which effectively improved the dehazing, denoising and visibility of underwater images [10]. 2. Zhou et al. proposed a method based on a color line model to generate underwater images with true color and natural appearance by filtering out image blocks with color line prior features [11]. 3. Song et al. proposed a systematic underwater image restoration method that estimates underwater light through quadtree refinement and optimizes the transmission map to enhance contrast [12]. 4. Li et al. proposed an underwater image enhancement algorithm based on an improved dark channel prior algorithm to solve the problem of too dark or too bright areas in the image [13]. The above methods mainly use underwater image restoration technology to focus on inversely solving the image degradation problem caused by light absorption and scattering in the underwater environment, thereby restoring the original clarity and color information of the image. However, these methods can only achieve satisfactory results when the underwater scene conforms to the selected model. Although the goal is clear, it limits the robustness and scalability of the model in complex and changing situations. In contrast, underwater image enhancement is more concerned with improving the observability of the image, focusing on processing the degraded image itself, and can retain and emphasize the visual details, color information, texture information and overall structure in the image, making the image more informative and visually effective, and has strong generalization ability and can handle diverse underwater environments. In summary, in order to improve the visual effect of underwater images, we propose a novel underwater image enhancement method.

### 2.2. CNN-Based UIE Methods

With the rapid development of convolutional neural networks, image restoration methods based on convolutional neural networks have achieved certain results, and more and more researchers have begun to apply convolutional neural networks to underwater image enhancement. The success of these methods is attributed to their powerful feature extraction capabilities and carefully designed network architectures, which can extract coarse-grained and fine-grained features in different receptive fields. For example, Anwar et al. [14] proposed the UWCNN network, which effectively improved the denoising and reconstruction performance of low-quality images by introducing a combination of super-resolution and convolutional neural networks. They also constructed a comprehensive underwater image dataset based on the physical model of underwater imaging and different water quality conditions for training the proposed underwater image convolutional neural network. In addition, Chen et al. [5] proposed an underwater image enhancement method based on deep convolutional neural networks, which effectively restored the color and details of the image by learning the local and global features of underwater images.

In order to better encode the global information of the image, the current research goal is to explore more powerful deep learning models. Most convolutional neural networks (CNNs) are good at extracting local features, and underwater images are enhanced using convolutional neural networks. Although convolutional neural networks can effectively achieve high accuracy, it is difficult for convolution to model global long-range relationships, which seriously limits the development of underwater image enhancement tasks. In contrast, the recently emerging Transformer model has shown significant advantages in capturing long-range dependencies and is therefore widely considered to be an effective way to improve the performance of image processing and enhancement tasks.

### 2.3. Transformer-Based UIE Methods

In addition to the traditional CNN architecture, in recent years, Transformer-based architectures have also been gradually applied to underwater image enhancement tasks. Transformer has shown significant advantages in capturing long-range dependencies and modeling global information, and has, therefore, become an important technology for enhancing underwater images. For example, as a new type of visual Transformer, Swin Transformer adopts a windowed self-attention mechanism, which can effectively improve computational efficiency while retaining global information, which has obvious advantages in underwater image enhancement tasks [15]. In addition, the UNet architecture has been widely used in underwater image enhancement. UNet can efficiently extract local features of images and maintain spatial information through a symmetrical encoding-decoding structure. The U-TransCNN model proposed by Y et al. [16] introduces the Transformer module into the encoder of UNet, making full use of the local feature extraction capability of CNN and the global information modeling advantage of Transformer, significantly improving the restoration effect of underwater images. Chen et al. [17] also proposed a hybrid model combining Transformer and convolutional neural networks to process information in underwater images. This method effectively improves the enhancement effect of underwater images under different water conditions by combining convolution and self-attention mechanisms. Although Transformer can improve the performance of the model, its own mechanism will bring a lot of GPU memory consumption and time overhead.

Therefore, our goal is to design a novel and more efficient Transformer for underwater image restoration based on the integration of CNN. At present, the dual-branch structure that can retain local features and global representation to the greatest extent is still temporarily shelved. The Dual-branch Feature Capture Block (DFCB) module is not only integrated into our MixRformer model through the dual-branch structure, but the MixRformer also pays more attention to details and global features and increases the model receptive field while keeping the computational complexity unchanged, reducing the number of model parameters. DFCB will be explained in detail in Section 3.3.

### 2.4. Wavelet-Based UIE Methods

Wavelets are widely used in underwater image enhancement (UIE) tasks. With the rise of deep learning, some studies have combined wavelets with CNN and achieved excellent results. For example, Wang et al. [18] designed a multi-level wavelet-based underwater image enhancement network (MWEN), combining a multi-level wavelet transform with color compensation prior to enhance underwater images. Subsequently, Zhao et al. [19] proposed a wavelet–Fourier information interaction with frequency diffusion adjustment (WFII-FDA), introducing a wavelet–Fourier information interaction module and a frequency residual diffusion adjustment module to enhance the high-frequency and low-frequency components of underwater images. Wang et al. [20] introduced a wavelet pixel domain progressive fusion network, which includes a wavelet domain module (WDM) to extract multi-scale frequency features with fine-grained details, thereby improving the quality of underwater images. Inspired by these methods, we intend to explore the performance of Transformers in the wavelet domain and build a more lightweight Transformer model using wavelets.

### 2.5. Style and Keypoint Learning-Based UIE Methods

In the field of underwater image processing, in addition to direct enhancement and feature extraction methods, style contrast learning and keypoint-sensitive learning provide important perspectives for related tasks. Recently, Wang et al. (2025) [21] proposed the content style control network (CSC-SCL), which is based on the CycleGAN framework. It separates content and style features through content control normalization (CCNorm) and style control normalization (SCNorm) and combines style contrast learning to improve the quality of underwater image enhancement, achieving higher indicators on the UIEB dataset. This method performs well in improving visual quality and domain generalization. On the other hand, Chen et al. (2024) [22] proposed hierarchical progressive learning (HTPL-JB), which optimizes feature extraction in sonar image classification through jigsaw self-supervised learning, hierarchical pre-training, and progressive training based on block convolution, combined with key point sensitive loss (KPSLoss), solves the problems of few samples and long-tail distribution, and achieves a G_mean of 94.77% on the NKSID dataset. Although the two methods focus on enhancement and classification, respectively, which are different from the underwater image enhancement task of this study (based on wavelet decomposition and dual-branch Transformer-CNN architecture), their feature processing strategies provide useful ideas for this field.

## 3. Proposed Approach

In this section, we first present the details of the proposed MixRformer model architecture, which is designed for underwater image enhancement with good robustness. Next, we elaborate on the key components of the network and finally introduce the loss function used for training.

### 3.1. Network Architecture

Our main goal is to develop a model that combines the characteristics of Transformer and CNN for underwater image enhancement in the wavelet transform domain by effectively co-optimizing the low- and high-frequency features in the wavelet transform domain. In computer vision tasks, the low frequencies in the wavelet transform domain mainly contain the main structure and basic texture information of the image, while the high frequencies correspond to the edges and detail features of the image. Low- and high-frequency information plays an important role in image restoration. Transformer and convolutional neural networks (CNNs) each have their own advantages. Transformer is good at capturing global dependencies and long-range relationships, but its computational complexity is high, which limits its application in high-resolution images. On the contrary, the CNN efficiently extracts detail features through local convolution operations, with low computational overhead, and is particularly suitable for processing edges and textures in images.

**Figure 1:**
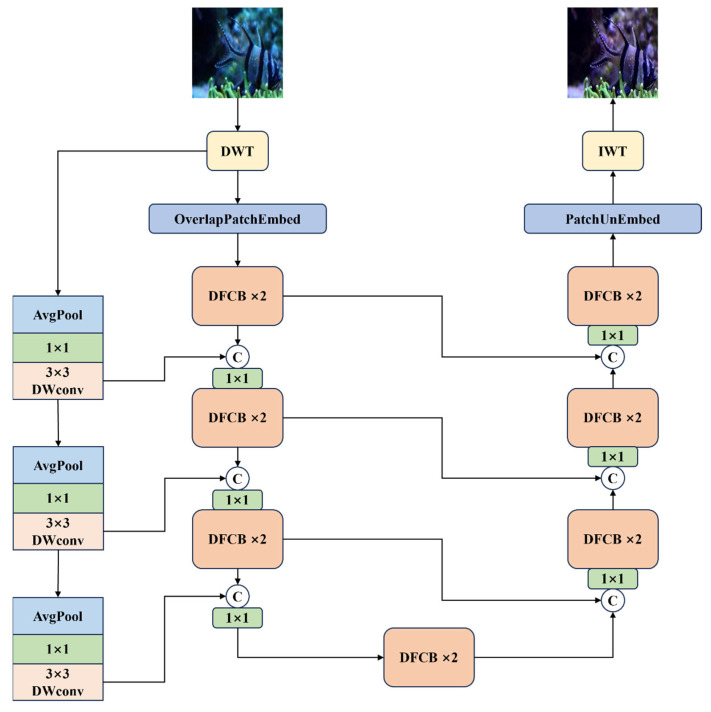
The overall architecture of MixRformer. It mainly combines wavelet transform with DFCB and coarse skip connections to process the information of underwater images in the frequency domain. The components will be introduced one by one later.

Therefore, combining Transformer with CNN can take advantage of the advantages of both: Transformer captures global information, and CNN extracts surface features. This combination not only improves the performance of the model, but also optimizes computational efficiency. As shown in Figure 1, the model MixRformer proposed in this study mainly consists of four parts: discrete wavelet transform (DWT), dual-branch feature extraction module (DFCB), inverse wavelet transform (IWT) and coarse skip-connection. Specifically, the model is built on a U-shaped architecture [23]. First, because the resolution of the input image is large, the model downsamples the input image through DWT, effectively extracting the high and low frequency information of the image while reducing the image resolution.

First, due to the large resolution of the input image, the model samples the input image through DWT, effectively extracting the high-frequency and low-frequency information of the image while reducing the image resolution, and concatenates the low-frequency and high-frequency four frequency domain image information to form a new input feature map, so that the feature map contains low-frequency and high-frequency information. The main part of the model uses the DFCB feature extraction module to perform feature processing on the new feature map after concatenation. We use DFCB to replace the pure convolution operation in the U-shaped structure, where the encoder gradually extracts the features of the input image through convolution layers and pooling layers and reduces the spatial resolution. On the other hand, at each stage of the encoding stage, our model sets a coarse skip connection. The role of this connection is to supplement the original features of the input data. It consists of a series of operations, including average pooling, pixel-level convolution, and depth convolution. Such a setting enables the encoder to focus more effectively on learning the residual caused by the absorption of light by water. In the encoder part, we give the network input as $I_{C}\in H\times W\times C$, In order to extract the wavelet domain information of the image and obtain low and high frequency information, DWT downsampling $h_{DWT}$ converts the input picture $I_{C}$ into four wavelet images:

(1) $$I_{LL},I_{LH},I_{HL},I_{HH}=h_{DWT}(I_{C})$$

where $I_{LL},I_{LH},I_{HL},I_{HH}\in \frac{H}{2}\times \frac{W}{2}\times C$ are 4 sub-images with different frequencies. Then, we connect the four sub-bands into a shallow feature $F_{e}\in \frac{H}{2}\times \frac{W}{2}\times 4C$. In order to fuse the low-frequency and high-frequency information, $F_{e}$ is subjected to a convolution operation for adaptive learning to fuse the features of each frequency and divide them into patches. These features are sent to the encoder composed of DFCB to further extract more effective features. In order to preserve the integrity of the image and reduce distortion, each layer of the encoder is combined with the shallow features of the upper layer through a coarse skip layer. When the network enters the bottleneck part, the bottleneck layer provides richer feature support for the decoder through global feature extraction and information compression to improve the enhancement effect. This part consists of only two dual-branch feature extraction modules. The decoder part is used for image reconstruction and adopts the same structure as the encoder part, retaining the dual-branch feature extraction module. Finally, in order to restore the image to the RGB channel, the IWT operation is used to convert the features to the original resolution and reconstruct the restored image.

### 3.2. Wavelet-Based Image Enhancement

In the image enhancement task, the low-frequency and high-frequency information in the image is a very important key factor for image restoration. The low-frequency sub-band contains the main structure and overall contrast information of the image, while the high-frequency sub-band corresponds to details such as edges and textures. At the same time, due to the large resolution of the general input image, the computational cost of the model for processing large-resolution images will be greatly increased. In order to solve the above problems, many methods have been proposed so far, such as convolution or pooling operations. However, some of these operations will cause irreversible information loss in the image, which is not conducive to image enhancement processing. Therefore, in order to solve the above problems, we introduced the wavelet transform into the model, which reduced the resolution of the image while introducing low-frequency and high-frequency information, greatly reducing the inference time and computational cost of the model.

**Figure 2:**
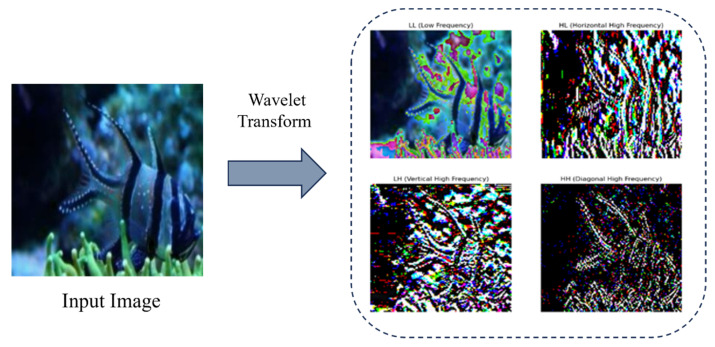
Schematic diagram of a wavelet transform.

Multi-scale frequency domain feature extraction [24] plays an important role in image enhancement tasks. As shown in Figure 2, the input image is subjected to multi-resolution analysis using a discrete wavelet transform (DWT), which can be decomposed into four sub-band components with different spatial frequency characteristics. A large number of studies and experiments have shown that the sub-bands decomposed by wavelet carry multi-dimensional feature representations of the image: the low-frequency component (LL) retains the global structure of the original image as a basic approximate representation; the vertical high-frequency component (HL) and the horizontal high-frequency component (LH), respectively, record the edge contour features in the corresponding directions; and the diagonal high-frequency component (HH) contains important oblique texture details. Combining features of different frequencies with the deep model of the Unet architecture can effectively guide the network to focus on visual features of different scales, thereby enhancing the model’s ability to restore image texture.

The innovative advantages of the wavelet transform we have made are as follows: specifically, the wavelet transform can be completely reversible for images and achieve lossless reconstruction of image information; secondly, the resolution of feature maps can be effectively reduced by multi-level the wavelet transform, which can greatly reduce the consumption of computing resources compared with traditional reduction methods, and does not introduce additional trainable parameters, significantly improving the efficiency of model reasoning; thirdly, sub-band components have dual representation capabilities of spatial position and frequency domain features, which is conducive to the network capturing geometric structure features in different directions; overall, the multi-scale characteristics of wavelet functions can expand the receptive field of the network, and on this basis, the multi-level feature fusion mechanism is used to enhance the model’s ability to express complex textures, providing an effective physical prior guidance for the model.

### 3.3. Dual-Branch Feature Capture Block

According to most previous Transformer-based methods, we found that they simply use the convolutional layer network for feature aggregation or sampling. However, the computational complexity of the Transformer itself is too large, which not only consumes a lot of computing resources but also causes the model performance to decrease due to multiple stacking of Transformers.

(2) $$Attention(Q,K,V)=Softmax(Norm(QK^{T}))V$$

**Figure 3:**
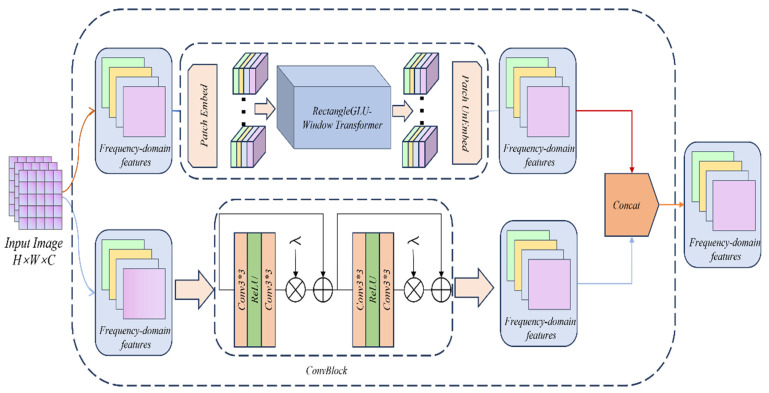
Detailed structure diagram and data flow of DFCB.

At present, CNN has been used as the backbone network in image processing. Its ability to extract local features and image location information is innate. Compared with Transformer’s artificial location encoding ability, it is far from CNN’s ability to automatically learn location information. However, Transformer can directly model global features through self-attention, allowing each pixel to pay attention to all pixels in the entire image. At the same time, Transformer itself can adaptively pay attention to information of different scales. Like CNN, it has its own natural multi-scale modeling ability, especially in dealing with complex details. Therefore, considering the multi-scale modeling ability, we found that introducing a multi-branch structure into the model can better extract visual features of different granularities. The multi-branch structure adopts a parallel structure to extract different features without interfering with each other. This design can avoid the problem of information sparsity caused by deep network stacking. As shown in Figure 3, our DFCB uses CNN and Transformer as two different branches, as surface information extraction branch and global information extraction branch, respectively. When shallow features are input into DFCB, they will enter both branches at the same time. Specifically, the surface information extraction branch contains a lightweight ConvBlock, which is a simple module consisting of two convolutional layers and a ReLU activation function. It introduces local residual connections to enhance feature reuse while retaining the surface information of the image. In the global information extraction branch, we designed an RGLUWin Transformer module to expand the attention receptive field without increasing the amount of computation, thereby maintaining the suppression or retention of information.

#### Rectangle GLU-Window Transformer Block

**Figure 4:**
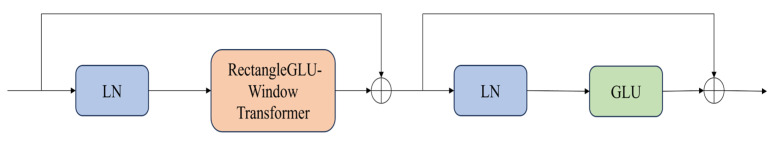
Block diagram of the RGLUWin Transformer.

The RGLUWin Transformer module is a key part of our dual-branch feature extraction module. As shown in Figure 3, the RGLUWin Transformer is used to extract the global context information of the image. It is more suitable for image restoration than the previous Transformer structure. Because the Transformer enables all image blocks to interact through global dependency modeling capabilities, its complexity is quadratic with the resolution of the image. Although local window self-attention reduces the computational complexity, the fixed division limits the cross-window interaction, resulting in limited receptive field expansion and insufficient modeling capabilities, affecting image restoration performance. For the above problems, we adopt a rectangular window self-attention mechanism, which is divided into a horizontal rectangular window (H-Rwin) and a vertical rectangular window (V-Rwin), and used in parallel for different attention heads. By aggregating features across different windows, the attention area is expanded without increasing the computational complexity, and the rectangular window self-attention is realized. In addition, the rectangular window can capture the different features of each pixel in the horizontal and vertical directions and aggregate the features of different windows to expand the receptive field, so that the model can build a differentiated receptive field, while the traditional square window is difficult to take into account the multi-scale and multi-directional feature expression requirements. The RGLUWin Transformer is shown in the Figure 4.

**Figure 5:**
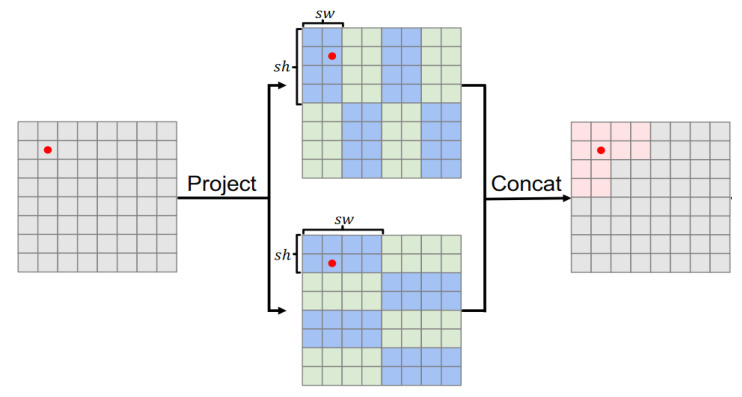
Rectangular Window Self-Attention Mechanism.

The special RGLUWin Transformer uses a rectangular window as shown in the Figure 5. (sh≠sw) instead of square windows (sh=sw), where sh and sw represent the height and width of the rectangle, respectively. In addition, we divide the rectangular window into horizontal windows (sh<sw, denoted as H-Rwin) and vertical windows (sh>sw, denoted as V-Rwin), and use them for parallel computation of different attention heads. In general, M attention heads are performed in parallel on the input feature $X\in R^{H\times W\times C}$. For each attention head, $X\in R^{H\times W\times C}$ is split into non-overlapping sh*sw rectangular windows, and the I-th rectangular window feature is represented as$\;X_{i}\in R^{(sh\times sw)\times C}$ where $i=1,,,\frac{H\times W}{sh\times sw}$ then the self-attention of the m-th head can be calculated as follows:

(3) $$\begin{array}{l} ({Q_{i}}^{m},{K_{i}}^{m},{V_{i}}^{m})=({X_{i}W_{m}}^{Q},{X_{i}W_{m}}^{K},{X_{i}W_{m}}^{V}),\\ \;\;\;\;\;\;\;Y_{i}^{m}=Attention({Q_{i}}^{m},{K_{i}}^{m},{V_{i}}^{m})=SoftMax(\frac{{Q_{i}}^{m}({K_{i}}^{m}{)}^{T}}{\sqrt{d}}+B){V_{i}}^{m} \end{array}$$

where $Y_{i}^{m}\in R^{(sh\times sw)\times D}$ is the attention feature of $X_{i}$ in the *m*-th head, ${Q_{i}}^{m},{K_{i}}^{m},{V_{i}}^{m}$ represent the projection matrices of the query, key and value of the head, respectively, $D=d=\frac{C}{M}$ is the channel dimension of each head, and *B* is the dynamic relative position encoding. Performing attention operations on all $X_{i}(i=1,,,\frac{H\times W}{sh\times sw})$, and reshaping and merging in the partitioning order, we can obtain the attention feature $Y^{m}\in R^{H\times W\times D}$ of the entire *X*.

Assuming that the number of attention heads M is even, the attention heads are evenly divided into two parts, and H-Rwin is performed on the first part and V-Rwin is performed on the second part. The outputs of the last two parts are concatenated according to the channel dimension. The calculation formula of this process can be expressed as follows:

(4) $$Rwin-SA\left(X\right)=Concat\left(Y^{1},Y^{2},\ldots ,Y^{M}\right)W^{p}$$

Among them, $Y^{1},\ldots ,Y^{\frac{M}{2}}$ are output using the H-Rwin head, and $Y^{\frac{M}{2}+1},\ldots ,Y^{M}$ are output using the V-Rwin head. $W^{p}\in R^{C\times C}$ represents the projection matrix of feature fusion.

**Figure 6:**
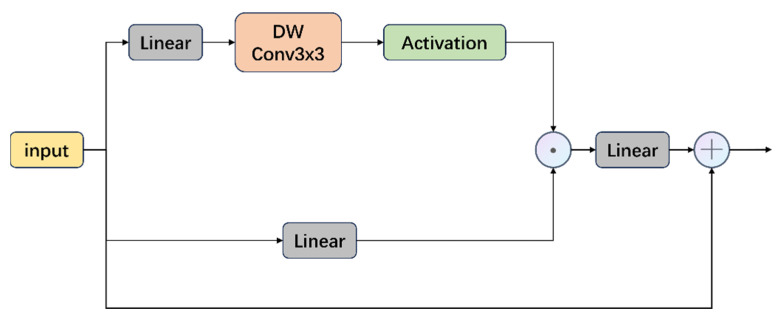
Detailed structure diagram of GLU.

At the same time, we found that the traditional MLP layer has inevitable shortcomings for underwater images. The GELU in the MLP will truncate the data, resulting in information loss. It also lacks selective information suppression or retention, and is not sufficient for position encoding. Inspired by [25], as shown in Figure 6 each token in the ConvGLU has a unique gating signal based on its most recent fine-grained feature. The gating mechanism suppresses the noise channel and strengthens the transmission of important features. At the same time, by introducing the 3 × 3 deep convolution, not only the shortcomings of the excessive coarse-grained global average pooling in the SE mechanism are solved, but also the deep convolution can provide position encoding. The 3×3 deep convolution retains spatial locality and makes up for the shortcomings of the Transformer in fine-grained texture modeling. In addition, the value branch of this design still maintains the same depth as the MLP and GLU, which is friendly to its back-propagation. Its calculation expression can be expressed as follows:

(5) $$ConvGLU\left(X\right)=\left(XW_{1}\right)⊙GELU\left(DWConv\left(XW_{2}\right)\right)$$

Among them, DWConv is a depth-wise separable convolution, and ⊙ represents element-by-element multiplication.

In general, the RGLUWin Transformer block we proposed helps the model capture the global information of the image through a rectangular window, and combines it with the GLU module to enable the model to retain useful information or suppress interference information in long-dependency information.

### 3.4. Loss Function

To train our model, we formulate a multinomial loss function which consists of two parts.

#### 3.4.1. MSE Loss

The mean square error loss is one of the basic functions in regression tasks. The model error is quantified by calculating the squared average of the difference between the generated image ${\hat{I}}_{j}$ and the reference image $I_{j}$. Its mathematical expression is as follows:

(6) $$L_{MSE}=\frac{1}{N}\sum_{i=1}^{N}{\left({\hat{I}}_{j}-I_{j}\right)}^{2}$$

#### 3.4.2. VGG Perceptual Loss

The calculation of mean square error loss is to optimize the model by the difference of pixel values. Excessive pursuit of pixel alignment may lead to overly smooth results and lack of details. Compared with mean square error loss, content-aware loss extracts high-level features through a pre-trained deep network (VGG) to measure the similarity between the generated content and the target in the feature space. In this chapter, a pre-trained 19-layer VGG network is used as a feature extractor [26], and the perceptual loss function is defined with a ReLU activation layer. Specifically, let $\phi_{j}\left(x\right)$ be the jth convolutional layer (after activation) of the VGG19 network ϕ pre-trained on the ImageNet dataset. The perceptual loss function is constructed by calculating the distance between the enhanced image $I_{e}$ and the reference image $I_{g}$ in the feature space, and its mathematical expression is as follows:

(7) $$L_{VGG}=L_{j}^{\phi }=\frac{1}{C_{j}H_{j}W_{j}}\sum_{i=1}^{N}∥\phi_{j}\left(I{’}_{e}\right)-\phi_{j}\left(I{’}_{g}\right)∥$$

where N represents the number of samples in each batch during training; $C_{j}H_{j}W_{j}$ represent the number, height and width of the feature map of the jth convolutional layer in the VGG19 network, which together define the dimension of the feature map.

In general, the total loss function can be expressed as follows:

(8) $$L_{TOIAL}=\lambda_{1}L_{MSE}+\lambda_{2}L_{VGG}$$

The coefficients are the weights of each loss, and we empirically set $\lambda_{1}$ and $\lambda_{2}$ to 1 and 1.

## 4. Experiments

### 4.1. Implementation Details

#### 4.1.1. Datasets

In this paper, to train and verify the generalization ability and effectiveness of MixRformer, we use the public underwater image dataset EUVP (https://irvlab.cs.umn.edu/resources/euvp-dataset) (accessed on 20 May 2025) [27]. This public dataset contains a large number of real underwater images and is divided into two subsets: paired data and unpaired data. The main advantages of the EUVP dataset are data diversity and strong restoration of real scenes. The images in this dataset are collected from different water environments, covering a variety of underwater environments (e.g., different turbidity, lighting conditions, water body types, etc.), and are shot using professional equipment, which better reflects the various challenges in real underwater imaging. At the same time, the dataset contains tens of thousands of images, which is very friendly to models containing Transformer and can reduce the risk of overfitting. This paper uses a paired dataset to train 11,435 images, of which 1270 images are used for verification and 515 images are used for testing. At the same time, in order to verify the generalization ability of our model, we use the 120 images in the test part of the public dataset UFO-120 (https://irvlab.cs.umn.edu/resources/ufo-120-dataset) (accessed on 20 May 2025) for generalization experiments. Experimental results show that our proposed model is superior in underwater image enhancement.

#### 4.1.2. Experimental Settings

Our network was implemented on Ubuntu 20 with Intel (R) Xeon E7-4809 v3 CPU, NVIDIA RTX 3090 GPU and PyTorch 3.10.6 framework. The model was trained for 100 epochs. We used Adam optimizer to train the model. To quantitatively compare the quality of enhanced underwater images, we used full-reference evaluation and no-reference evaluation metrics to measure the performance of different methods. We considered two commonly used full-reference evaluation standard metrics, namely Peak Signal-to-Noise Ratio (PSNR) and Structural Similarity (SSIM) [28], and three commonly used no-reference evaluation metrics, namely Underwater Image Quality Metric (UIQM), Underwater Image Color Quality Evaluation Metric (UCIQE) and Natural Image Quality Evaluation Metric (NIQE) to quantify and evaluate the color and structural similarity between MixRformer enhanced images and their corresponding real images. Generally, higher values of PSNR, SSIM, UIQM and UCIQE indicate better performance, and lower values of NIQE indicate better performance.

### 4.2. Comparative Experiments and Analysis

To demonstrate the performance of the proposed model, in this section we compare the MixRformer model with the following ten competitive underwater image enhancement models, which cover a variety of methods, including BRUE [29], based on Retinex variational theory, UDCP [4] and IBLA [30], based on physical models, ShallowUWnet [31] and Uice2Net [32], based on convolutional neural networks (CNNs), UGAN [33] and FunieGAN [34], based on generative adversarial networks (GANs), and U-Trans [35], combining GAN and Transformer. In addition, two advanced models that fuse CNN and Transformer are also included, namely AutoEnhancer [36] and Frequency [37]. By comparing with these representative models, the performance advantages of MixRformer are comprehensively evaluated.

#### 4.2.1. Qualitative Evaluation

**Figure 7:**
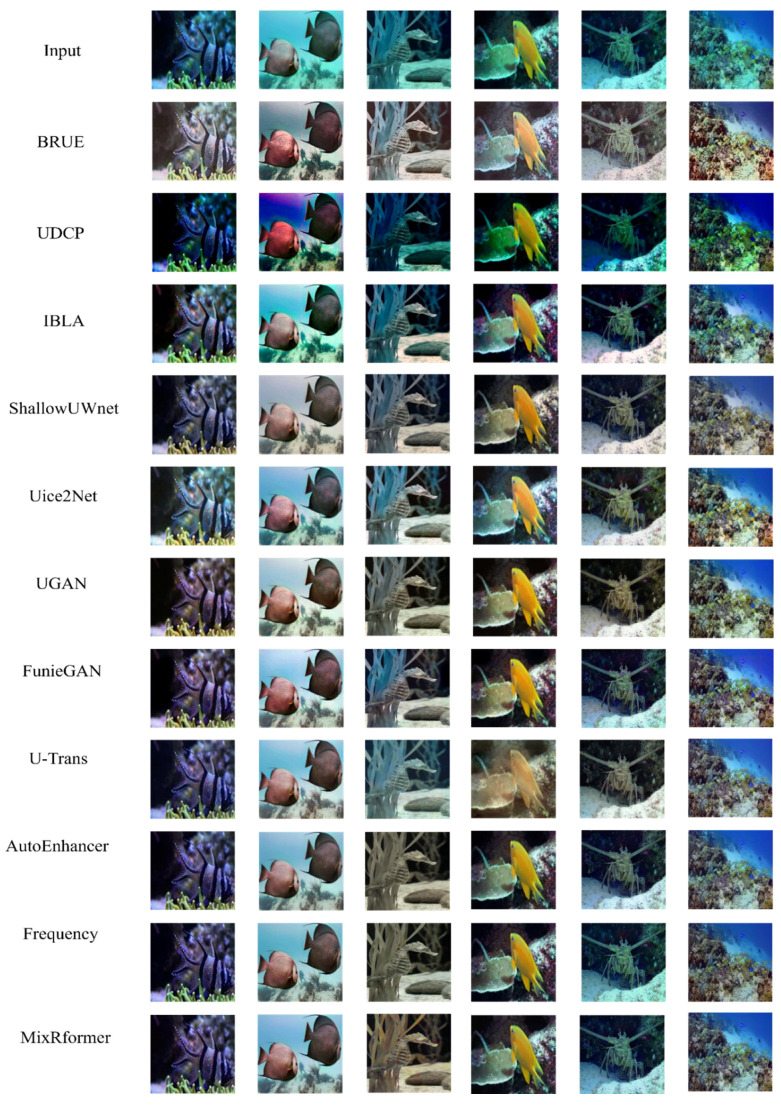
Qualitative comparison results of MixRformer and other methods on EUVP.

Figure 7 is the result obtained through qualitative evaluation. Specifically, each model enhances underwater images to varying degrees, but the results are unsatisfactory.

**Figure 8:**
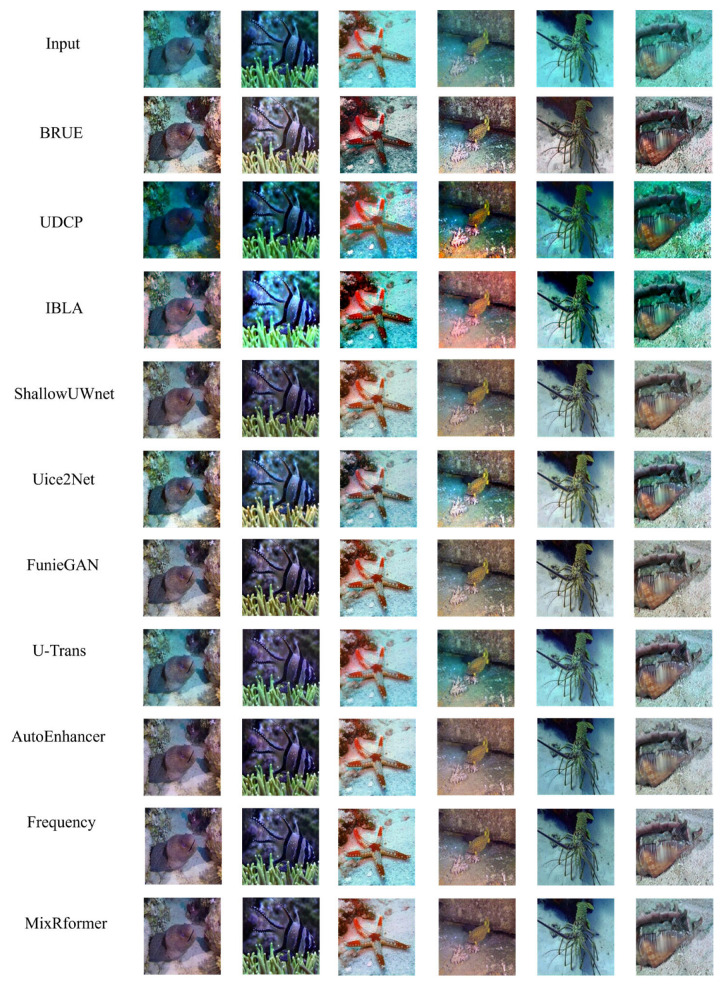
Qualitative comparison results of MixRformer and other methods on UFO-120.

Figure 8 shows the results of qualitative evaluation on the UFO-120 dataset to demonstrate generalization ability. It can be seen that each model has different degrees of generalization to enhance underwater images, but the effect is not ideal.

It can be seen that BRUE, based on non-physical models, cannot specifically restore distorted underwater images, and the restored images show exposure and color distortion. UDCP and IBLA are based on physical models; although the former can improve the brightness of the red channel to a certain extent, it cannot restore distorted underwater images, the background of the image deepens the blue-green tone, and the image details and textures are greatly lost. Due to the limitations of light propagation characteristics, the latter still shows a blue-green bias after enhancement, and the local contrast is over-enhanced. ShallowUWnet and Uice2Net are models based on CNN architecture. Compared with previous models, it is obvious that they have improved in effectively correcting local color deviation and enhancing texture details. However, there is an overall lack of brightness, the imbalance of color control in local areas leads to overexposure, and the plants in the picture are not green, but enhanced in yellow, which eventually leads to color deviation. After the emergence of GAN networks, UGAN and FunieGAN, the background image is no longer dark overall, and the enhancement effect is gradually closer to the real image. From the effects shown in Figure 1 and Figure 5, it can be found that due to the severe scattering in the underwater environment, the image generated by UGAN is yellowish, the highlight part has overexposure artifacts, and the edge is distorted; FunieGAN, as can be clearly seen from Figure 3 and Figure 5, can significantly improve the edge clarity of the image. The color contrast of the background in the figure is most similar to our Mixformer. However, like UGAN, the color of the seaweed in Figure 1 is still yellow. The U-Trans network has unstable training factors. Compared with the previous model, Figure 1 significantly restores the green color of the seaweed, but the hazy part in Figure 4 makes the enhanced image blurry. The AutoEnhancer and Frequency algorithms that aggregate CNN and Transformer perform well in visual effects. The images enhanced by visual perception are excellent in high-frequency texture sharpness and low-frequency color fidelity, which are comparable to this method, but the former still has excessive yellowing in some areas in detail texture restoration.

In addition, we use the UFO-120 test set to qualitatively verify the generalization ability of this model. BRUE has high color saturation and contrast, which can effectively enhance the visual impact of the image, but the color is easy to deviate from the sense of reality. UDCP performs well in enhancing the blue-green tone and restores the details well, but some images have slight over-sharpening. IBLA is still the algorithm with the worst processing effect and fails to effectively remove the blue-green bias. Shallow-UWnet, Uice2Net, FunieGAN, and U-Trans still cannot effectively solve the color bias problem. The images processed by the first three models are obviously yellowish, and U-Trans is no longer yellowish, but the excessive increase of green tones makes the image too bright; however, they are worthy of recognition in restoring image details. AutoEnhancer, Frequency and our model, MixRformer, can effectively improve image quality and restore image color, but AutoEnhancer has deficiencies in processing image brightness, and Frequency has certain color distortion problems, but our model achieves very similar results.

In contrast, we can see that by using our Mixformer to obtain low-frequency and high-frequency information through wavelet transformation and dual-branch feature extraction module, the enhanced image is not only closer to the reference image in terms of detail texture and color restoration, but also has a more moderate contrast and more natural color performance.

#### 4.2.2. Quantitative Evaluation

In addition to the qualitative evaluation, we also conducted a comprehensive evaluation of the performance of the model on the EUVP dataset in the quantitative evaluation. It can be clearly seen from Table 1 that our MixRformer achieved the best results in terms of PSNR, SSIM and NIQE evaluation indicators compared with other competing methods, which shows that MixRformer has significant effects in enhancing the detailed texture of underwater images, restoring the structural similarity of images, and enhancing the visual characteristics of images closer to natural images. From the various indicators obtained, it can be seen that the underwater images enhanced by MixRformer not only have a good improvement in contrast, but the model also has a good correction effect on the color shift of underwater images relative to the reference image. In order to verify the generalization ability of the model, we compared our model and the competitive methods in the test set of the UFO-120 dataset. It can be seen from Table 2 that the results clearly show that our model still achieves the best in terms of PSNR, SSIM and NIQE evaluation indicators, indicating that MixRformer can effectively enhance images in different underwater environments.

### 4.3. Ablation Study

We use a series of ablation experiments to prove and evaluate the effectiveness of each component in Mixformer.

**Figure 9:**
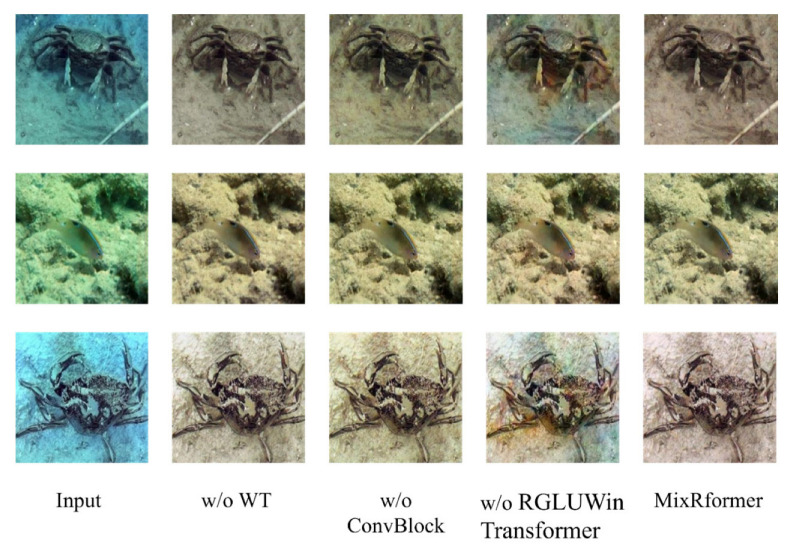
The figure shows the qualitative ablation study of WT, ConvBlock, and RGLUWin Transformer, respectively.

In our model, the most important ones are WT, ConvBlock and RGLUWin Transformer. In order to verify the effectiveness of each of these blocks, we have performed many ablation experiments to prove it. The WT operation we introduced plays a vital role in shortening the execution time, GPU memory consumption and introducing wavelet information. In order to verify that WT can do these things, we conducted a comparative experiment. From the results in Table 3, it can be seen that when we introduce WT, MixRformer only uses one-fourth of the running time and one-fourth of the GPU memory occupancy to achieve a significant reduction in the amount of calculation and complexity. At the same time, it can be seen in Figure 9 that the overall image without WT is not bright enough and the color is dark. The w/o ConvBlock block basically retains the local features of the image, such as texture, but the contrast of the image is not obvious and the edge information is too blurred. The w/o RGLUWin Transformer block does not effectively eliminate the blue-green tones of the image, and the local area is occupied by blue-green tones. The importance of WT to the model is also clearly seen from the quantitative analysis in Table 4. MixRformer is improved by one point under the effect of WT. In general, compared with our MixRformer which controls the information flow through GLU, ConBlock for surface information, and RGLUWin Transformer for fine-grained extraction, on the basis of reducing resource consumption, the enhanced image not only retains the local detail texture, but also basically eliminates the color deviation in the image, enhancing the visual perception and contrast effect.

**Figure 10:**
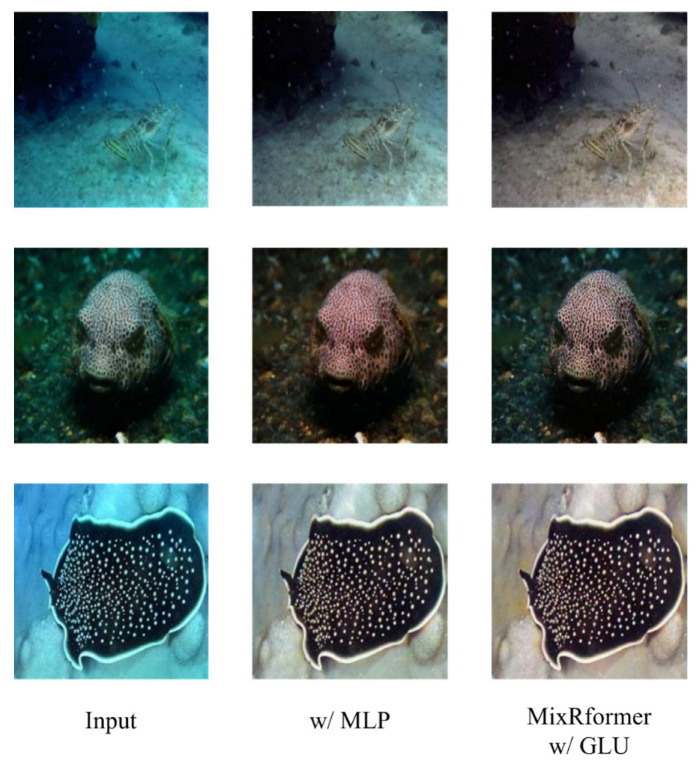
Qualitative comparison between MLP and GLU.

For the introduced GLU module, we conducted a qualitative and quantitative comparison between GLU and MLP on RWin Transformer. As shown in Figure 10, when using the traditional MLP, the removal of blue-green tones in Figure 1 and Figure 3 is far inferior to the effect of w/GLU. At the same time, from the results of Figure 2, it can be observed that the contrast of marine animals and the sharpness of texture and detail restoration on the animals are all enhanced by GLU better than MLP. At the same time, from the quantitative aspect, it can be found from Table 5 that GLU is stronger than MLP in both information suppression and selection.

**Figure 11:**
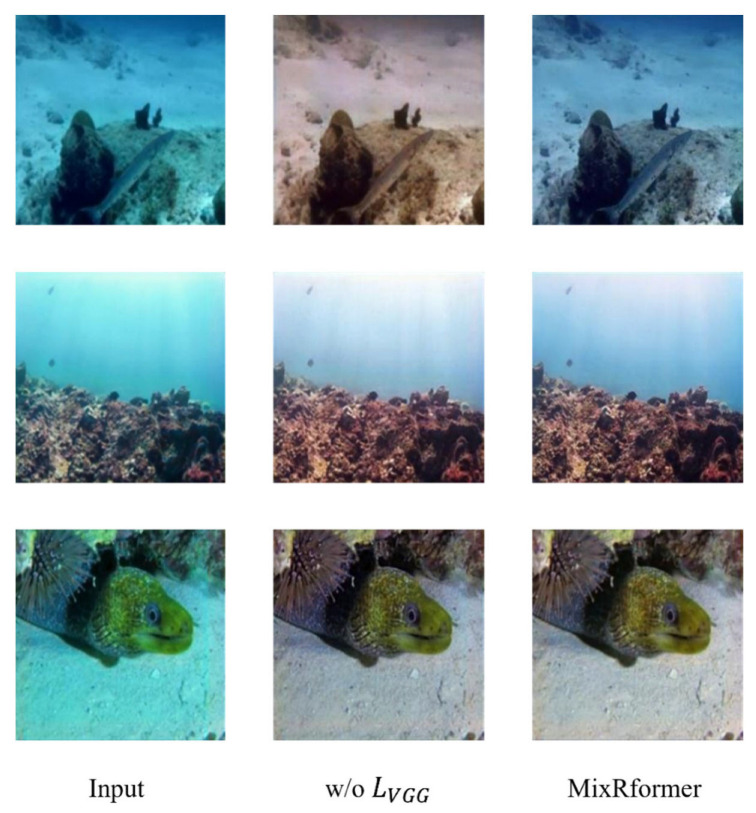
Qualitative ablation experiments on each of the multiple loss functions.

Of course, in order to verify the effectiveness of the components of the multi-loss function we set in achieving high-quality image restoration, we conducted an ablation experiment on the loss function. It can be clearly seen from Figure 11 that when w/o VGG, the image restoration is also relatively clear, and the texture details are gradually restored. However, the image is obviously reddish, and the contrast and generalization ability are reduced, and the color calibration is offset, as in Figure 1 and Figure 2. In contrast, using a complete multi-loss function helps to provide more comprehensive and fine texture details and a contrast closer to the reference image. It can be clearly seen from the data in Table 6 that when using a multi-loss function, the PSNR and SSIM indicators are significantly improved in value, so that the network can restore and enhance the image from multiple angles, and achieve enhanced generalization ability for complex underwater environments.

At the same time, in order to verify the effectiveness of wavelet transformation on the number of parameters and FLOPs of our method, we compared the number of parameters occupied by different modules in our method. The specific results are shown in Table 7. It can be clearly seen that when we introduce wavelet transformation, without basically increasing the number of model parameters, it not only greatly reduces the FLOPs of the model, but also improves the visualization effect of the image and improves the quantitative evaluation index. This shows that MixRformer, while reducing FLOPs, obtains satisfactory visual effects and quantitative indicators.

## 5. Conclusions

In this paper, we propose the MixRformer model, a novel method for underwater image enhancement. In detail, we introduce the wavelet transform into our model, which uses low-frequency information for image enhancement, restores the texture and detail information of the image, and greatly reduces the image resolution, reduces GPU memory consumption, and reduces computational cost. At the same time, this paper proposes a dual-branch feature extraction module (DFCB) that combines the advantages of CNN and Transformer to fully restore image details by extracting local and global features. In addition, we also design multiple loss functions based on the traditional loss function to meet multiple constraints and realize the model’s adaptability to complex environments. Extensive ablation experiments show that the important components of our MixRformer improve the model performance both quantitatively and qualitatively, and the model execution time and GPU memory consumption have been significantly improved. In the future, we will better optimize MixRformer for tasks related to underwater video enhancement.

## Figures and Tables

**Table 1:** The following table shows the results of quantitative comparison between our model and various current representative models on EUVP. The bold font represents the result with the highest index.

Model	PSNR	SSIM	UIQM	UCIQE	NIQE
BRUE	17.58	0.67	3.17	0.42	4.92
UDCP	15.84	0.57	2.00	0.52	5.66
IBLA	16.55	0.43	2.25	0.49	5.50
ShallowUWnet	26.75	0.79	2.91	0.398	6.04
Uice2Net	26.83	0.86	2.99	0.396	5.83
UGAN	25.42	0.81	2.94	0.41	5.15
FunieGAN	26.24	0.81	2.89	0.45	5.66
U-Trans	22.57	0.74	3.00	0.38	5.04
AutoEnhancer	28.78	0.86	2.85	0.41	5.06
Frequency	28.81	0.86	2.82	0.42	4.95
Ours	**29.01**	**0.87**	2.88	0.42	**4.916**

PSNR, Peak Signal-to-Noise Ratio; SSIM, Structural Similarity; UIQM, Underwater Image Quality Measurement; UCIQE, Underwater Color Image Quality Evaluation; NIQE, Natural Image Quality Evaluator.

**Table 2:** The following table shows the results of a quantitative comparison between our model and various current representative models on UFO-120. The bold font represents the result with the highest index.

Model	PSNR	SSIM	UIQM	UCIQE	NIQE
BRUE	17.05	0.64	**3.** **09**	0.43	4.96
UDCP	19.12	0.66	2.25	**0.51**	5.11
IBLA	20.06	0.71	2.45	0.49	5.06
ShallowUWnet	27.02	0.81	2.89	0.43	5.16
Uice2Net	21.00	0.75	3.02	0.45	5.03
FunieGAN	26.97	0.83	2.73	0.45	4.88
U-Trans	25.29	0.80	2.96	0.42	4.99
AutoEnhancer	28.13	0.84	2.79	0.43	4.88
Frequency	28.33	0.84	2.73	0.44	4.91
Ours	**28.43**	**0.** **85**	2.84	0.43	**4.68**

PSNR, Peak Signal-to-Noise Ratio; SSIM, Structural Similarity; UIQM, Underwater Image Quality Measurement; UCIQE, Underwater Color Image Quality Evaluation; NIQE, Natural Image Quality Evaluator.

**Table 3:** Comparison of WT model usage on GPU. The bold font represents the result with the highest index.

Model	Patchsize	GPU	Time/epoch
w/o WT	3	21,976 MiB	66 m
MixRformer	**12**	**23,418 MiB**	**17 m**

**Table 4:** Quantitative Ablation Study on WT, ConvBlock, and RGLUWin Transformer. The bold font represents the result with the highest index.

Model	PSNR	SSIM	UIQM
w/o WT	28.083 ± 3.369	0.835 ± 0.062	2.859 ± 0.388
w/o ConvBlock	28.521 ± 3.08	0.847 ± 0.054	2.845 ± 0.393
w/o RGLUWin Transformer	28.59 ± 3.163	0.846 ± 0.052	2.88 ± 0.371
MixRformer	**29.01 ± 3.158**	**0.87 ± 0.054**	2.88 ± 0.387

PSNR, Peak Signal-to-Noise Ratio; SSIM, Structural Similarity; UIQM, Underwater Image Quality Measurement.

**Table 5:** The following table shows a quantitative comparison of MLP and GLU. The bold font represents the result with the highest index.

Model	PSNR	SSIM	UIQM
w/MLP	28.89 ± 3.222	0.854 ± 0.053	2.873 ± 0.402
MixRformer w/GLU	**29.01 ± 3.158**	**0.87 ± 0.054**	**2.88 ± 0.387**

PSNR, Peak Signal-to-Noise Ratio; SSIM, Structural Similarity; UIQM, Underwater Image Quality Measurement.

**Table 6:** The following table is a quantitative ablation study of the loss function.

Model	PSNR	SSIM	UIQM
w/o *L_VGG_*	28.669 ± 3.136	0.85 ± 0.052	2.841 ± 0.412
MixRformer w/GLU	**29.01 ± 3.158**	**0.87 ± 0.054**	**2.88 ± 0.387**

PSNR, Peak Signal-to-Noise Ratio; SSIM, Structural Similarity; UIQM, Underwater Image Quality Measurement.

**Table 7:** The following table is a quantitative comparison of the number of parameters and FLOPs.

Model	Parameters	FLOPs
w/o ConvBlock	10.10 M	11.58 G
w/o RGLUWin	12.97 M	15.29 G
Transformer	20.05 M	100.16 G
w/o WT		
MixRformer	**20.06 M**	**25.17 G**

## Data Availability

The data are from public datasets, which are introduced in Section 4.1.
